# Rapid Discrimination of *Salmonella enterica* Serovar Typhi from Other Serovars by MALDI-TOF Mass Spectrometry

**DOI:** 10.1371/journal.pone.0040004

**Published:** 2012-06-29

**Authors:** Martin Kuhns, Andreas E. Zautner, Wolfgang Rabsch, Ortrud Zimmermann, Michael Weig, Oliver Bader, Uwe Groß

**Affiliations:** 1 Institute for Medical Microbiology and International Health Network Göttingen, University Medical Center Göttingen, Göttingen, Germany; 2 German National Reference Center for Salmonella and other Enteric Pathogens, Robert Koch Institute, Wernigerode, Germany; Indian Institute of Science, India

## Abstract

Systemic infections caused by *Salmonella enterica* are an ongoing public health problem especially in Sub-Saharan Africa. Essentially typhoid fever is associated with high mortality particularly because of the increasing prevalence of multidrug-resistant strains. Thus, a rapid blood-culture based bacterial species diagnosis including an immediate sub-differentiation of the various serovars is mandatory. At present, MALDI-TOF based intact cell mass spectrometry (ICMS) advances to a widely used routine identification tool for bacteria and fungi. In this study, we investigated the appropriateness of ICMS to identify pathogenic bacteria derived from Sub-Saharan Africa and tested the potential of this technology to discriminate *S. enterica* subsp. *enterica* serovar Typhi (*S*. Typhi) from other serovars. Among blood culture isolates obtained from a study population suffering from febrile illness in Ghana, no major misidentifications were observed for the species identification process, but serovars of *Salmonella enterica* could not be distinguished using the commercially available Biotyper database. However, a detailed analysis of the mass spectra revealed several serovar-specific biomarker ions, allowing the discrimination of *S.* Typhi from others. In conclusion, ICMS is able to identify isolates from a sub-Saharan context and may facilitate the rapid discrimination of the clinically and epidemiologically important serovar *S*. Typhi and other non-*S.* Typhi serovars in future implementations.

## Introduction

Fever is a leading cause for hospital admission in Sub-Saharan Africa. Often malaria is thought to be the underlying disease; however bacterial blood stream infections (BSI) contribute to a significant proportion of febrile illness [1], [2], [3], [4]. Bacterial BSI is an important cause of morbidity and mortality [5], and in case of septic shock mortality can be as high as 60% [6]. It was well demonstrated that time between onset of septic shock and start of adequate therapy is essential for survival [7]. However, as microbiological diagnostics is often not available in African countries due to infrastructure, budget and personnel constraints, clinicians have to rely on syndrome-based empirical approaches to treat febrile illness [8], [9]. Consequently, fever in Africa is often treated sequentially: first with anti-malarial drugs and then with antibiotics, risking poor clinical outcome and development of resistance [10], [11].

Whereas *Staphylococcus aureus* and *Escherichia coli* continue to be the most common causative agents of true BSIs in developed countries [12], Gram-negative bacteria, in particular *Salmonella enterica*, are the main cause of BSI in African countries [3], [13], [14]. Both, *S. enterica* serovar Typhi (*S*. Typhi) and non-typhoid *Salmonella* are frequently isolated from blood cultures in African countries [15] and typhoid fever, caused by *S.* Typhi, is estimated to annually cause about 21 million cases and approximately 217.000 deaths [16]. Varied manifestations of typhoid fever are observed especially in pediatric patients, including septicemia, diarrhea and lower respiratory tract infections [17]. In the sub-Saharan regions of Africa multidrug-resistant typhoid fever (MDRTF) is becoming a serious problem [17]. Since the 1980s repeated outbreaks with MDRTF associated with increased morbidity and mortality have been reported, particularly in malnourished children and children below an age of five years [17]. The MDRTF rate in Kenya increased from 5% to 77.2% within 1988–2008 [18], [19] and is similar in Nigeria 61% [20] and Ghana 63% [21].

The laboratory diagnosis of *S. enterica* relies on bacterial culture using different selective media [22]. As most *S. enterica* strains produce hydrogen sulfide with exception of *S.* Paratyphi A and some *S.* Typhi strains, they can generally be distinguished from other *Enterobacteriaceae* using thiosulfate containing agar (10). However, some species like *Citrobacter freundii* are also able to produce hydrogen sulfide and differentiation between *S. enterica* and *C. freundii* can therefore be challenging. Consequently, various other chromogenic media have been developed to discriminate between *C. freundii* and *S. enterica* [22], [23], [24]. For further subtyping of *S. enterica* the White-Kauffmann-Le Minor classification scheme [25] or phage typing [26] are in use, however the latter technologies are not available in most laboratories.

**Table 1:** Concordance of species identification by conventional and ICMS methods.

Species	N°	conventional ID	ICMS – ID
*Enterobacteriacae*			
*Citrobacter freundii complex*	*3*	ok	ok
*Enterobacter cloacae complex*	*9*	ok	ok
*Escherichia coli*	*10*	ok	ok
*Klebsiella oxytoca*	*1*	ok	ok
*Klebsiella pneumoniae*	*6*	ok	ok
*Klebsiella pneumoniae* a	*1*	*Enterobacter spec.*	ok
*Klebsiella variicola* a	*1*	*Pantoea agglomerans*	ok
*Proteus mirabilis*	*3*	ok	ok
*Proteus vulgaris*	*1*	ok	ok
*Providencia rettgeri*	*1*	ok	ok
*Salmonella ssp.*	*160*	ok	ok
*Shigella flexneri* a	*1*	ok	*Escherichia coli*
gram+ cocci			
*Enterococcus casseliflavus*	*1*	ok	ok
*Enterococcus faecalis*	*3*	ok	ok
*Lactococcus lactis* a	*1*	*Enterococcus faecium*	ok
*Staphylococcus aureus*	*26*	ok	ok
non-fermenter			
*Achromobacter xylosoxidans*	*1*	ok	ok
*Acinetobacter baumanii complex*	*8*	ok	ok
*Acinetobacter junii*	*1*	ok	ok
*Acinetobacter junii* a	*1*	*Acinetobacter haemolyticus*	ok
*Acinetobacter junii* a	*1*	*Acinetobacter lwofii*	ok
*Chryseobacterium gleum* a	*1*	*Chrysobacterium indologenes*	*Chryseobacterium indologenes*
*Comamonas aquatica* a	*1*	*Achromobacter xylosoxidans*	*Comamonas testosterone*
*Pseudomonas aeruginosa*	*17*	ok	ok
*Pseudomonas putida complex*	*1*	ok	ok
*Psychrobacter pulmonis* a	*1*	*Pseudomonas stutzeri*	*Psychrobacter ssp.*
*Stenotrophomonas maltophilia*	*2*	ok	ok
gram+ rods			
*Brevibacterium casei* a	*2*	*Rhodococcus equi*	ok
*Microbacterium arborescens* a	*1*	*Corynebacterium ssp.*	ok
*Paenibacillus spp.* a	*1*	no reliable identification	no reliable identification
*Rhodococcus pyridinivorans* a	*1*	*Corynebacterium ssp.*	*Rhodoccus rhodochrous*
*Sinomonas flava* a	*1*	*Brevibacterium ssp.*	*Sinomonas atrocyanea*

a In the case of absence or discordance of identifications by conventional and ICMS, the correct species was identified by sequencing of the 16S rDNA locus.

Currently, intact cell mass spectrometry (ICMS) advances to a widely used routine identification tool for bacteria and fungi [27],[28]. Here, mass spectra from whole bacterial or fungal cell lysates are used for identification [27]. This method was previously shown to identify Salmonellae at the species and subspecies level [29]. Additionally, it was shown that serovar-specific biomarker ions can be found in ICMS spectra allowing the distinction of *S*. Enteritidis, *S*. Typhimurium/4, 5, 12:i:-, *S*. Virchow, *S*. Infantis, *S*. Hadar, *S*. Choleraesuis, *S*. Heidelberg, and *S*. Gallinarum. However, the clinically most important serovar *S.* Typhi was not included in those particular analyses [29], [30].

In this study, we used blood culture isolates taken from a study population suffering from febrile illness in Ghana [3] and additional *S. enterica* reference strains to investigate the suitability of ICMS to (i) identify pathogenic bacteria derived from sub-Saharan Africa (spectrum databases were generated with isolates originating mainly in the Western World) and (ii) test the potential of this technology to discriminate *S.* Typhi from other serovars.

## Materials and Methods

### Strains

Isolates used for our analyses were taken from a previous epidemiologic study done in three independent locations in Ghana [3]. In that study, isolates were obtained from blood cultures of patients with fever of unknown origin and differentiated by conventional means (microscopy, API systems, agglutination with antisera using the White-Kauffmann-LeMinor scheme). Further *Salmonella S*. Typhi subtyping was done with the Vi phage typing scheme [31]. To exclude a bias towards potential clonal outbreaks in the Ghanaian study centers, we included 44 additional pseudonymized isolates obtained and archived during routine diagnostic procedures in Göttingen or the Salmonella Reference Center.

In total, our set contained 160 *Salmonella enterica* subsp. *enterica* isolates of 12 different serovars (84x *S*. Typhi, 51x *S*. Typhimurium, 14x *S*. Enteritidis, 2x *S*. Typhimurium var. Copenhagen 2x *S*. Paratyphi, one each of serovars Albany, Brandenburg, Infantis, Hadar, Tennessee, and two not further characterized non-*S.* Typhi serovars) as well as other species present in the blood cultures (Table 1), as described previously [3].

**Figure 1:**
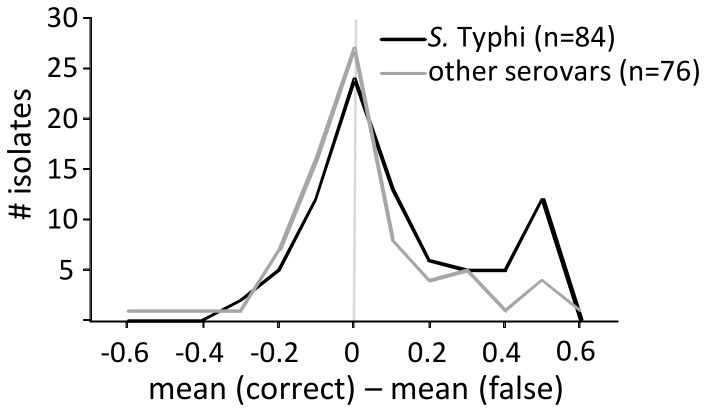
Value of BioTyper hit scores for *S.* Typhi identification. Negative values indicated higher false hit scores, positive values indicated higher correct hit scores. A value near zero indicated a similar score distribution between correct and false hits.

### Intact Cell Mass Spectroscopy

Cells were grown over night on sheep blood agar (Oxoid, Wesel, Germany) at 37°C under safety conditions as required, prepared in duplicate for ICMS by smear preparation and overlaid with HCCA matrix, both under a safety cabinet and after drying transported to the MALDI device. ICMS was done by standard procedures recommended for the BioTyper 3.0 system (Bruker Daltonics, Bremen, Germany). For analysis, 600 spectra from 2–20 kDa were gathered in 100-shots steps. Results with score values >2.000 were considered correct. Analyses for isolates not yielding a significant score were repeated once by smear preparation and in the case of 22 (all non-Salmonella) isolates subsequently by formic acid-acetonitrile extraction. All ICMS identification experiments were done in a blinded form. In 15 cases 16 S rDNA sequencing was used as a tie-breaker for discordant or unclear results.

At the time of investigation, the Biotyper 3.0 and SR databases together contained 29 spectra from the genus *Salmonella* (*S.* Typhi (10), *S.* Paratyphi (3), S. *enterica* subsp. *Arizonae* (2), and one spectrum each of *S*. *Bongori*, *S*. Anatum, *S.* Choleraesuis, *S. enterica* subspec. *diarizonae*, *S.* Dublin, *S.* Enteritidis, *S.* Gallinarum, *S.* Hadar, *S. enterica* subspec. *houtenae*, *S. enterica* subspec. *indica, S. enterica* subspec. *salamae*, *S*. Stanley, *S*. Typhimurium, and one untyped). For the global identification procedure all of these were counted as “*Salmonella enterica* spp.”. Phyloproteomic analyses were done using Flexanalysis and PCA algorithms implemented into the BioTyper 3.0 software (both Bruker Daltonics). Spectra were pre-processed by baseline subtraction and smoothing, for PCA-based hierarchical clustering distance measurement was set to ‘correlation’; the linkage algorithm to ‘average’.

**Figure 2:**
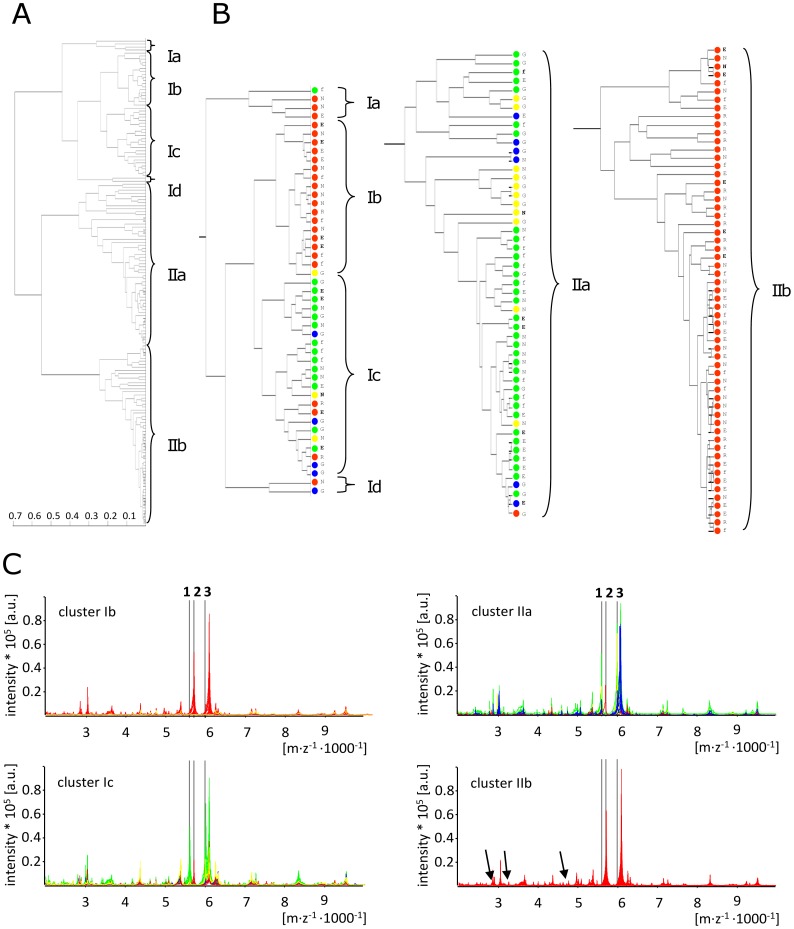
Relatedness of *Salmonella enterica* ICMS spectra reflects serotype. (A) Global cluster analysis of *S. enterica* isolates. (B) Enlargement of major clusters from (A). Serovars: *S*. Typhi (red), *S.* Typhimurium (green), *S*. Enteritidis (yellow), others (blue). Isolate sources: G:Göttingen; R:Salmonella Reference Center; E:Eikwe; N:Nkawkaw; f:Fosso. Isolation time points in Ghana (E, N, and f only): not bold  = 2006; bold  = 2009 (C) Overlay of ICMS spectra contained in the four major clusters identifies at least one major peak (peak 2; m/z  = 5713.9) specific to *S*. Typhi (red) and two major peaks (peaks 1 and 3; m/z = 5616.7 and m/z = 6009.7 respectively) specific for non-*S*. Typhi isolates (green, yellow and blue). Several other small peaks specific for *S*. Typhi were also seen (three example peaks indicated in cluster IIb by arrows, m/z = 2856.4, m/z = 3258.0, and m/z = 4716.3, respectively).

## Results

### Classification Results

A total of 225 blood culture isolates plus 44 *S. enterica* control strains were re-typed using ICMS (Table 1). No major misidentifications were observed. Where discordant results were obtained, ICMS identifications were either correct or at least phylogenetically closer to the species eventually identified by 16 S rDNA sequencing, with the exception of one *Shigella flexneri* isolate. Discrimination of *S. enterica* spp. from other Enterobacteriaceae including *Citrobacter* spp. (as well as other genera) was 100%.

To analyze the usability of Biotyper score values for the discrimination of *S.* Typhi from other *Salmonella* serovars, score values for all *Salmonella* isolates were obtained for all *S. enterica* spp. spectra contained in the database and a “delta mean score” (geometric mean [correct hits] - geometric mean [false hits]) calculated (Figure 1). Due to the lack of multiple spectra for each of the different serovars in the database, all non-*S*. Typhi isolates were considered as one group and all *S*. Typhi isolates as the other. This analysis showed that spectra from *S*. Typhi isolates did not reproducibly give higher score values with *S*. Typhi database entries. Although a certain number of spectra, for which most high ranking hits were correct, were observed, false hits were always present with scores >2.000. Similarly, non-*S*. Typhi isolates also produced high score values with *S.* Typhi database entries.

### Phyloproteomic Analysis of *Salmonella* Isolates

To further determine whether the different *Salmonella* serovars can be differentiated by their ICMS-spectra, the spectra were clustered and the phyloproteomic nearness was analyzed. Surprisingly, the three major *Salmonella* serovars tested (Typhi, Enteritidis and Typhimurium) clustered into several well-separated groups (Figure 2A). With only five outliers (5.9%), *S*. Typhi isolates fell into only two distinct sets (Figure 2B, clusters 1b and 2b). This clustering was independent of the isolate origin, indicating that this nearness did not reflect a clonal outbreak (Figure 2B). A correlation of the *S*. Typhi clusters with the Vi phage type was not observed; however this may have been missed as the vast majority of the isolates (75%) were of phage type D1 (data not shown). In an overlay of spectra from the four major clusters at least three major and several smaller peaks can be identified, which separate *S*. Typhi isolates from other serovars (Figure 2C). These differences in biomarker ions separating *S*. Typhi from other serovars were present independently of the cluster the spectrum was contained in.

## Discussion

Today, the laboratory diagnosis of *S.* Typhi is predominantly based on the White-Kauffmann-Le Minor classification scheme [25] or phage typing [31] following bacterial culture. Although there are several approaches to substitute bacterial culture and SV determination by PCR [32], these assays have a limited sensitivity and offer no substitution for antibiotic susceptibility testing [33] making culture-based approaches still indispensable.

In this context, MALDI-TOF MS-based ICMS has recently advanced to a widely used routine species identification tool for cultured bacteria and fungi [27], [28]. To analyze whether this method was also applicable to isolates from a sub-Saharan context, we retyped a previously established collection of Ghanaian blood culture isolates [3] by ICMS. This collection included a significant number of *S. enterica* isolates.

With the exception of one *Shigella flexneri* isolate, no clinically important errors were observed. Also, discrimination of *S. enterica* spp. from other Enterobacteriaceae was 100%. As demonstrated here and also by others [27], [28] species identification from ICMS spectra is very robust and generally only dependant on the presence of the respective spectrum in the database. As shown here, it is applicable not only to isolates obtained in developed countries, but also to countries from sub-Saharan Africa.

In contrast to species identification, subtyping within a single species (or differentiation between extremely close related species) is a more subtle process. In our study this was demonstrated by the inability of the system to discriminate *E. coli* and *S. flexneri* or to type inside the genus *Salmonella*. This lack of implementation is also officially stated by the manufacturer. Nevertheless, previous phyloproteomic analyses have shown spectrum clusters of *S*. Typhi isolates among other Enterobacteriaceae [34] and several biomarker ions that differentiate non-*S*. Typhi isolates from each other [30]. In our analysis, smear spectra obtained from *S.* Typhi isolates were of such difference from other serovars that they could be clustered into distinct sets. Furthermore, we were able to identify at least six biomarker ions that differentiate *S*. Typhi from non-*S*. Typhi spectra. Thus, we were able to discriminate *S.* Typhi from other *S. enterica* serovars using ICMS.

In conclusion, our study demonstrates that (i) ICMS-based species identification is applicable to isolates from sub-Saharan Africa and (ii) that it is possible to discriminate clinically important subtypes, such as the serovars inside the *S. enterica* subspecies even using smear spectra. This finding should be of special interest in areas where enteric bacteria, particularly *Salmonella enterica*, are highly prevalent as causative agent of BSI and other severe infections and together with new enrichment technologies [35], this should lead to significant speed increase in Salmonella diagnostics. Future research will therefore be directed to implement this in the respective commercial ICMS technologies using weighted pattern matching and specific reference spectra.
